# Narrowband Characterization of Near-Ground Radio Channel for Wireless Sensors Networks at 5G-IoT Bands

**DOI:** 10.3390/s18082428

**Published:** 2018-07-26

**Authors:** Hicham Klaina, Ana Vazquez Alejos, Otman Aghzout, Francisco Falcone

**Affiliations:** 1 Department of Signal theory and Communications, University of Vigo, 36310 Vigo, Spain; analejos@uvigo.es; 2 Department of Telecommunications, ENSA, LabSit-FS, Abdelmalek Essaadi University, 93030 Tetouan, Morocco; o.aghzout@gmail.com; 3 Department of Electronic and Electrical Engineering, Public University of Navarre, 31006 Pamplona, Spain; francisco.falcone@unavarra.es

**Keywords:** Internet of Things, 5G, wireless sensor networks, smart agriculture, near-ground, radio channel model, QoS, log-distance

## Abstract

In this contribution, a narrowband radio channel model is proposed for rural scenarios in which the radio link operates under near-ground conditions for application in wireless sensor networks dedicated to smart agriculture. The received power attenuation was measured for both transmitter and receiver antennas placed at two different heights above ground: 0.2 and 0.4 m. Three frequency ranges, proposed for future 5G-IoT use case in agriculture, were chosen: 868 MHz, 2.4 GHz and 5.8 GHz. Three ground coverings were tested in a rural scenario: soil, short and tall grass fields. The path loss was then estimated as dependent of the radio link range and a three-slope log-normal path loss model was tailored. Results are explained in terms of the first Fresnel zone obstruction. Commercial Zigbee sensor nodes operating at 2.4 GHz were used in a second experiment to estimate the link quality from the experimental Radio Signal Strength Indicator (RSSI) received values. Two sensor nodes were placed at the same elevation above ground as in the previous experiment, only for short grass field case. The Quality of Service performance was determined in terms of theoretical bit error rate achieved for different digital modulations—BPSK, 8PSK and 16QAM—concluding remarkable results for an obstructed radio link.

## 1. Introduction

Wireless Sensor Network (WSN) technology is based on collecting data from sensor nodes, which communicate between each other and between them and the gateway, which transmit the data through internet for storage, analysis and processing [1,2]. It has attracting applications in a variety of fields such as health care [3,4], means of land transport [5,6], machine surveillance, military uses and others [7].

In the past few years, the WSN has also emerged into the research field of agriculture, and it is a candidate application for the use cases proposed for Internet of Things in the fifth generation (5G) of wireless mobile communications [8]. This short-coming future of precision agriculture received the name of the fourth revolution [9] and Internet of Agriculture [10]. Among other benefits, the WSN technology provides a feasible way for low-cost, high-efficiency, and high-productivity agriculture farming [11,12]. Some agriculture related parameters, such as soil temperature, soil moisture, CO_2_, PH value and soil nutrients, could be monitored in real-time and then controlled by wireless sensor networks [13] even in an automated manner. The new scope of the smart agriculture will undoubtedly bring a wide variety of business opportunities.

However, the use of WSN technology in agriculture is not totally free of challenges. The first issue to overcome might be the fact that the nodes are usually quite close to the ground and sometimes even just on the ground, producing that the height of the antennas above ground is usually low and it may approach zero. This setup leads the radio link that the first Fresnel zone is obstructed in a different way to one introduced by an obstacle. The obstruction due to obstacles can turn the radio path into an obstructed line of sight (OLoS) or a non-line of sight (NLOS) radio link, and it has been largely modelled in literature for different scenarios, including vegetation [14].

Even that a formal definition has not been formulated, if the first Fresnel zone (FFZ) is obstructed along a distance larger than a critical value to not be considered a simple obstacle, it is assumed that the radio propagation operates under a close to ground or near-ground case.

The specific distance along which the FFZ is obstructed was modelled in [15,16] for millimeter frequency bands and depends on the antenna height, the frequency and the ground dielectric parameters. This critical distance $d_{C}$ is larger than the break point distance $d_{B}$ given by the two-ray propagation model, and whilst the path loss rate is 20 dB/decade before the break point it increases to 40 dB per decade after $d_{B}$. Both break and critical points are plausible to be present in a WSN even that the network configuration was not originally planned as a near-ground design. The break point is a function of the antenna height and frequency, and for a 0.5 m of antenna altitude operating at 2.4 GHz $d_{B}$ is about 20 m. Thus, if the two-ray model is assumed as valid to model the near-ground case, an underestimation of the path loss occurs at larger transmitting distances.

In [17] the first model discussing the inverse relation between antenna altitude and the received signal strength was introduced, even that not fulfilled the near-ground definition. There are few models proposed in literature for the near-ground channel: many only based on measurement campaigns, others are based on simulations not corroborated by experimental measurements and others were derived as mathematical models.

Most of the few existing models proposed in literature for the near-ground channel are incomplete and become specific to the considered scenario, frequency bands, antenna radiation pattern and polarization, terrain roughness and profile, ground composition and dielectric properties [15,16] or the fixed antenna height. Recently, near-ground wireless sensor networks are attracting increasing attention, especially for military applications [18,19,20,21,22]. A radio channel model for near ground agriculture applications is rarely considered among the latest literature.

Then, it is evident the need for reliable near-ground narrowband radio channel modelling which allows understanding of the undergoing propagation behavior. The path loss model becomes crucial for the design and the evaluation of robust wireless systems for smart agriculture applications. Moreover, it is to evaluate the maximum effective distance between adjacent terminals, hence to estimate the number of sensors needed to cover a certain area.

When placing nodes close to the ground in agriculture fields, the transmitted signal experiences a high-level of attenuation due to the components of the field like grass and soil, as well as terrain roughness. As we mentioned above, path loss caused by near-ground placement of antennas is mainly explained in terms of Fresnel zones obstruction [15,16,23,24,25]. This signal strength loss plays a key role on the quality of service (QoS) causing unreliable communication between nodes leading to an unsuccessful WSN application that will increase both the number of data packet retransmissions and the power consumption in the nodes, causing radio link failure in last term.

In this paper a narrowband radio channel model is proposed to predict path loss, in order to fit the signal strength decay caused by agriculture fields found in the propagation link for a near-ground radio scenario at microwave frequency bands. An experimental measurement campaign was carried out using identical transmitter and receiver directional antennas working at 868 MHz, 2.4 GHz and 5.8 GHz, and general purpose lab instrumentation, in agriculture fields with three types of ground: soil, short and tall grass. Firstly, the received power is measured at two different heights of 20 cm and 40 cm above ground. The path loss was then estimated as dependent of the radio link range and antenna height, introducing modifications according to the observations and results achieved.

Following, in a different scenario setup, analyzed was the communication quality between commercial sensor nodes dedicated to smart agriculture by measuring the Radio Signal Strength Indicator (RSSI) value as a quality indicator, when placing the nodes at the same two different antenna heights, 20 and 40 cm, only for the case of a short grass field. These nodes were working at 2.4 GHz under ZigBee protocol. From the RSSI measured values, the value of the bit error rate (BER) was derived for different transmitted signal modulation schemes (BPSK, 8-PSK, 16-QAM) in order to compare the theoretical performance of those modulations in a near-ground scenario.

In Section 2 are described the measurement systems used in the two experiments and the planned campaigns. Section 3 introduces the experimental results achieved for the three types of grounds and antenna heights. Section 3 also contains the analysis of the experimental results. The proposed path loss model, based on a log-distance fitting, is briefly described in Section 3. Section 4 elaborates a discussion for comparison between the achieved path loss model and results and other existing works. Finally, conclusions close this paper.

## 2. Materials and Methods

### 2.1. Measurement System for Determining Path Loss Model 

The objective of this experiment was to determine the path loss introduced by the ground interfering the FFZ in a single radio frequency carrier propagated between the transmitter and receiver when changing height, by lowering both antennas from $H_{1}$ = 40 cm to $H_{2}$ = 20 cm. Furthermore, we will see the difference in the path loss when using three different frequency bands $F_{1}$ = 868 MHz, $F_{2}$ = 2.4 GHz and $F_{3}$ = 5.8 GHz.

In the transmitter end, a power of $P_{t}$ = 6 dBm has been used. For 868 MHz, the frequency carrier was provided by the programmable synthesizer HM 8133-2 that generates frequencies less than 1 GHz. However, for 2.5 GHz and 5.8 GHz, the signal generator SRM40 has been used.

The received power was measured by spectrum analyzer FSH6 and recorded in a laptop using the software FSH View. An averaging of 10 is used to reduce the noise in the data traces.

**Figure 1:**
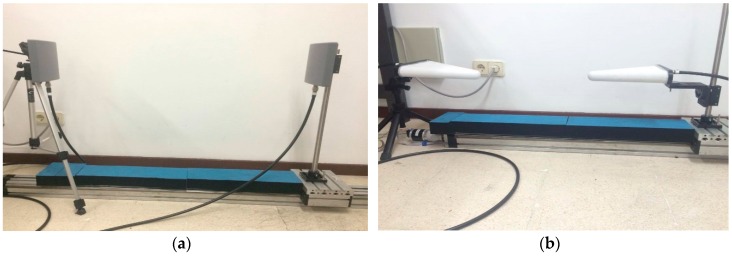
Path loss measurement system: (**a**) 868 MHz antenna setup; (**b**) 2.4/5.8 GHz antenna setup.

Identical omnidirectional antennas were used in the transmitter and receiver ends. The antenna model for the 868 MHz pilot was EAD SCP868-5 (gain = 5 dBi, E-plane = 75°, H-plane = 140°), and for the 2.4 GHz and 5.8 GHz pilots the antenna model was EM-6952 (gain = 4.41/4.71 dBi, E-plane = 70°, H-plane = 125°). Figure 1 depicts a test performed in the lab with both antennas to verify the system operation. We should notice that the flat panel antenna used for 868 MHz shows circular polarization, meanwhile the log-periodic antenna used for 2.4/5.8 GHz is linearly polarized.

### 2.2. Path Loss Measurement Campaign 

The first step was to fix both transmitter and receiver on a height of $H_{2}$ = 0.4 m from the ground. Measurements have been started with a separation distance of $d_{1}$ = 1 m. The transmitter was kept stationary and the receiver was moved each time with 2 m. At each receiver antenna position, the received power was measured and recorded. The next step was to lower the height of both transmitter and receiver simultaneously to $H_{1}$ = 0.2 m and apply the same procedure of the first step.

**Figure 2:**
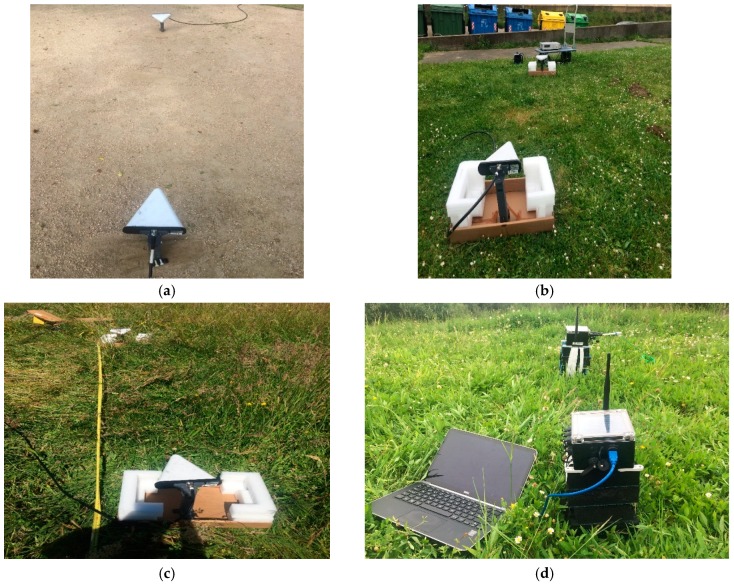
Detail of measurement campaigns: (**a**) soil field with *H*_1_; (**b**) short grass field with *H*_1_; (**c**) tall grass field with *H*_1_; and (**d**) Radio Signal Strength Indicator (RSSI) with waspmote nodes in tall grass field with *H*_2_.

The same described method was used for each of the three frequency carriers, $F_{1}$ = 868 MHz, $F_{2}$ = 2.4 GHz and $F_{3}$ = 5.8 GHz. Pictures in Figure 2 try to illustrate the experimental scenarios of the measurement campaign.

### 2.3. RSSI System Measurement 

The objective of this experiment was to gather the RSSI data in a short grass field to study how radio signal behaves in near ground conditions by using commercial WSN nodes model XBee-ZB-PRO operating at 2.4 GHz with ZigBee protocol. The elevation of the nodes was varied to observe the impact of the ground on the signal quality.

A radio link was established between two nodes: one was intended for transmitting data frames and the other one was for receiving and measuring the RSSI values. Both nodes are provided a standard stub antenna with omni-directional radiation pattern in the E-plane. The transmitted power was $P_{t}$ = 17 dBm, and the receiver sensitivity is −102 dBm.

The firmware of the nodes was programmed to ensure a CW transmission. RSSI was calculated at the radio chip on the base station in real-time and provides useful implication of wireless link quality [9]. Later, following the indications of the manufacturer, the link quality between nodes is estimated in percentage from the RSSI values, according to the following flowchart (1)–(3):

(1) if RSSI≥−50 dB then Quality = 100%

(2) else if RSSI≤−100 dB then Quality = 0%

(3) else Quality(%) ≈ 2×(RSSI(dB) + 100).

Initially, both nodes were fixed at a height where the elevation of the antenna was $H_{2}$ = 40 cm from the ground with $d_{1}$ = 1 m as separation distance between them. The transmitter node was kept stationary and the receiver node was moved each time a step of 2 m, until completing a final separation distance of 50 m. RSSI was measured at each position of the receiver. After that, the nodes were placed on the ground so the elevation of the antennas from the ground was $H_{1}$ = 20 cm, and the same procedure as the first one was repeated.

**Figure 3:**
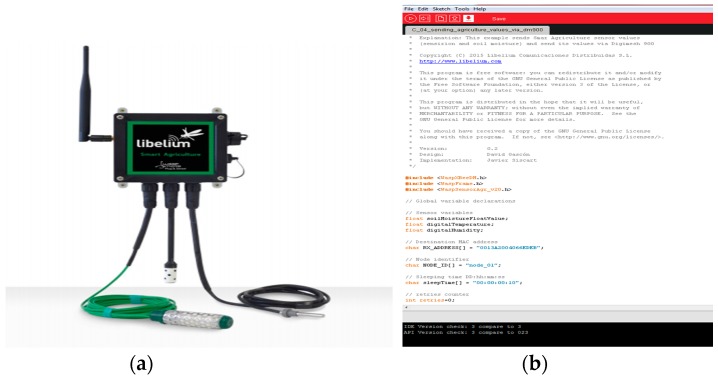
RSSI measurement system: (**a**) commercial Wireless Sensor Network (WSN) node used; (**b**) programming interface.

In Figure 3a, the sensor node XBee-ZB-PRO is shown. Figure 3b shows the programing interface used to modify the original firmware.

## 3. Results

### 3.1. Analysis of Experimental Results for Path Loss 

After finishing with measurements, path loss $P_{L}\left(d\right)$ was calculated, in dB, as the difference between the received power $P_{r}\left(d\right)$ measured at the point of distance d from the transmitter and the received power $P_{r}\left(d_{0}\right)$ at the first point $d=d_{0}=1\;m$, that is used for reference distance:

(4) $$P_{L}\left(d\right)=P_{r}\left(d\right)-P_{r}\left(d_{0}\right)\;[\mathrm{dB}]$$

**Figure 4:**
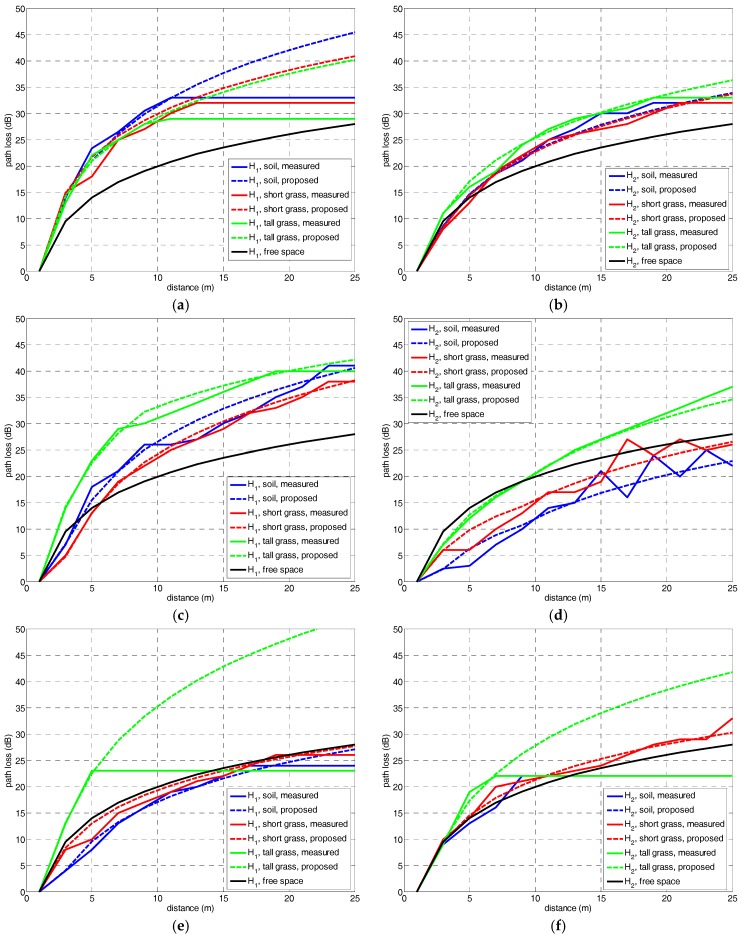
Path loss experimental results and comparison to free-space model and proposed model, at (**a**) $F_{1}$, $H_{1}$; (**b**) $F_{1}$, $H_{2}$; (**c**) $F_{2}$, $H_{1}$; (**d**) $F_{2},\;H_{2}$; (**e**) $F_{3},\;H_{1}$; and (**f**) $F_{3}$, $H_{2}$.

The $P_{L}\left(d\right)$ resulting for the combination of the three types of ground coverings—soil, short grass and tall grass—, three frequency bands—$F_{1}$ = 868 MHz, $F_{2}$ = 2.4 GHz and $F_{3}$ = 5.8 GHz—and the two antenna heights—$H_{1}=20\;\mathrm{cm}$ and $H_{2}=40\;\mathrm{cm}$—, are following shown in Figure 4. Results are compared to the free space path loss model. For better comparison, all the figures are depicted using the same range in the *y*-axis, from 0 to 50 dB.

Generally speaking, path loss values are increasing when lowering the antenna height. The difference between the free-space and measured path loss also decreases with the increase of the frequency range. This latter result is explained by the obstruction of the FFZ which presents a radio $r_{1}=\sqrt{d\lambda }=2.94\;m$, and remains permanently obstructed for all the cases. In Table 1 the clearance or obstruction of the FFZ is indicated in percentage with respect to the antenna height. The percentages indicate the existence of near-ground scenario for both $H_{1}$ and $H_{2}$ with FFZ obstruction over >60% that is considered the limit for the existence of losses due to diffraction (OLoS).

For $F_{1}$ = 868 MHz, the path loss is higher than that one due to the free-space model, and over the whole radio link distance. For $F_{2}$ = 2.4 GHz, the path loss seems to modify the trend with the antenna height likely due to a lesser FFZ obstruction. Finally, for $F_{3}$ = 5.8 GHz, the path loss is the case more similar to the free-space curve due to the fact that the FFZ is obstructed in a lower percentage ~65% for $H_{2}$ close to an obstruction-free condition (LoS).

The type of ground covering also seems to influence the path loss trend. Soil and short grass present almost the same path loss in most cases. This may be due to the short height of the grass.

However, the highest level of attenuation was experienced in tall grass field due to the length of the grass and the existence of more leafs that is reducing the clearance between the transmitter and the receiver, so lowering the effective antenna height to a value lower than $H_{1}$ and $H_{2}$.

### 3.2. Near Ground Path Loss Model

The following step is to determine the path loss trend to determine the most suitable modelling. In all the path loss curves it was detected that the path loss modifies its trend at two points, namely critical points: one close to the transmitter ($d_{C0}$) and a second one ($d_{Cf}$) shortly after the previous. These points derive from the obstruction of the first Fresnel ellipsoid and the plane of the ground and split the propagation distance into three zones:

- Zone #1: from the transmitter to the first critical distance $d_{C0}$. In this first stage, the 2-ray propagation becomes dominant; however, the power combination reaching the receiver results in path loss rate equal to free-space for $H_{2}$, or less restrictive for $H_{1}$.
- Zone #2: this region is placed between both critical points and remains under obstruction larger than 60%; is the very zone of the near-ground scenario; the path loss increases its rate.
- Zone #3: beyond the second critical point, it is observed a more destructive path loss rate, due to the combination in the receiver antenna of diffracted and scattered rays coming out from the zone #2. Once this last region ends, the path loss remains constant.

In order to determine the path loss trend, a log-distance fitting was applied to each of the three zones. The resulting path loss model represents the power attenuation, in dB, as a logarithmic function of the distance and the critical points occurrences and meets the expression given in (5)–(7):

(5) $$\begin{array}{cc} P_{L}\left(d\right)=10\cdot n_{1}\cdot \log_{10}(d), & \;d\leq \;d_{C0} \end{array}$$

(6) $$\begin{array}{cc} P_{L}\left(d\right)=10\cdot n_{2}\cdot \log_{10}(d), & \;d_{C0}\leq \;d\leq \;d_{Cf} \end{array}$$

(7) $$\begin{array}{cc} P_{L}\left(d\right)=10\cdot n_{3}\cdot \log_{10}(d), & d\geq \;d_{Cf} \end{array}$$

where the variables $n_{1}$, $n_{2}$ and $n_{3}$ indicate the attenuation factor in dB/m for each propagation region. The values of the attenuation factors are summarized in Table 2. A value of 2 indicates that the attenuation matches the free-space model.

The critical points $d_{C0}$ and $d_{Cf}$ are obtained as per (8):

(8) $$\begin{array}{cc} d_{C0}=\frac{{H_{1,2}}^{2}}{p\cdot \lambda } & ,\;d_{Cf}=2\cdot \;d_{C0} \end{array}$$

here $H_{1,2}$ is the height of the transmitter and the receiver antennas, λ is the wavelength of the transmitted wave, and p is the ratio $H_{1,2}/r_{1}$.

The free space path loss $L_{fs}$ is obtained in dB as per (4):

(9) $$L_{fs}=32.44\;+\;20\;\log_{10}\left(f\;\times \;10^{-6}\right)\;+\;20\;\log_{10}\left(d\;\times 10^{-3}\right)$$

where *f* is the transmitted signal frequency in Hz and *d* is the distance between the transmitter and the receiver in meters.

In Figure 4, the measured results were compared to the proposed three-slope log-distance model resulting in a close approach between experimental and theoretical values. The proposed path loss model also indicates that the power attenuation due to the free-space model is not realistic for a correct prediction.

### 3.3. RSSI Measurement Results

**Figure 5:**
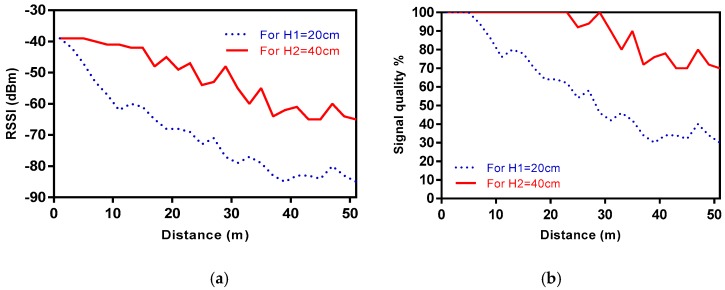
Difference at $H_{1}$ and $H_{2}$ between (**a**) RSSI values; (**b**) radio link quality.

Using commercial sensor nodes dedicated to smart agriculture, RSSI values were recorded in order to observe the impact of the short grass field under near ground conditions. Results are depicted in plots of Figure 5.

From Figure 5a it is noticed that the height of the sensors above the ground may have a significant impact not only on the signal strength but also on the signal quality. The achieved results indicate that the higher the position of the sensor above the ground, the stronger the signal strength becomes in accordance to the path loss results previously analyzed. The link quality indicator was estimated from the measured RSSI values using the flowchart given in (1)–(3), for both nodes elevations.

As shown in Figure 5b the link quality between the nodes improves for $H_{2}$. At this height, the signal quality remains at 100% until 29 m. However, at $H_{1}$ the signal quality is decreasing from 5 m. When the receiver node was placed 51 m away from the transmitter the signal quality is over 70% for $H_{1}$ whilst is under 35% at the same distance for $H_{2}$.

The observed results may be interpreted under the sight of a radio link provided of Obstructed-line-of-sight (OLoS). For such cases, a break point *d_B_* occurs defined as the location in which the FFZ contacts the ground, and it is calculated as per (10):

(10) $$d_{B}\approx \frac{4h_{tx}h_{rx}}{\lambda }$$

with *h_tx_* and *h_rx_* the transmitter and receiver antenna heights, and *λ* the wavelength. For a scenario with *H*_1_ antenna height, the break point distance corresponds to a distance of around 32 m from the transmitter.

### 3.4. Analysis of Quality of Service 

Following the Quality of Service (QoS) performance of our close-to-ground scenario, the 2.4 GHz case is analyzed. Bit Error Rate (BER) is a key parameter used for determining the QoS of wireless communication systems. BER expression for QPSK, *M*-PSK and *M*-QAM modulations can be calculated by (11)–(13):

(11) $$BER_{QPSK}=Q\left(\sqrt{2\frac{E_{b}}{N_{0}}}\right)$$

(12) $$BER_{MPSK}≅\frac{2}{\log_{2}M}Q\left(\sqrt{2\frac{E_{b}}{N_{0}}\log_{2}M}\cdot \sin\left(\frac{\pi }{M}\right)\right)$$

(13) $$BER_{MQAM}≅\frac{4}{\log_{2}M}Q\left(\sqrt{3\frac{E_{b}}{N_{0}}\cdot \frac{\log_{2}M}{M-1}}\right)$$

where *E_b_* = *P_Rx_*/*R_b_*, with *P_Rx_* the received power obtained for each spatial location along the pathway in the considered scenarios as RSSI measurements. The noise power can be estimated as *N*_0_ = *K*·*T*·*B*, with *K* the Boltzman’s contant, *T* the temperature (290 K), and *B* the frequency bandwidth of the 2.4 GHz and 868 MHz bands

**Figure 6:**
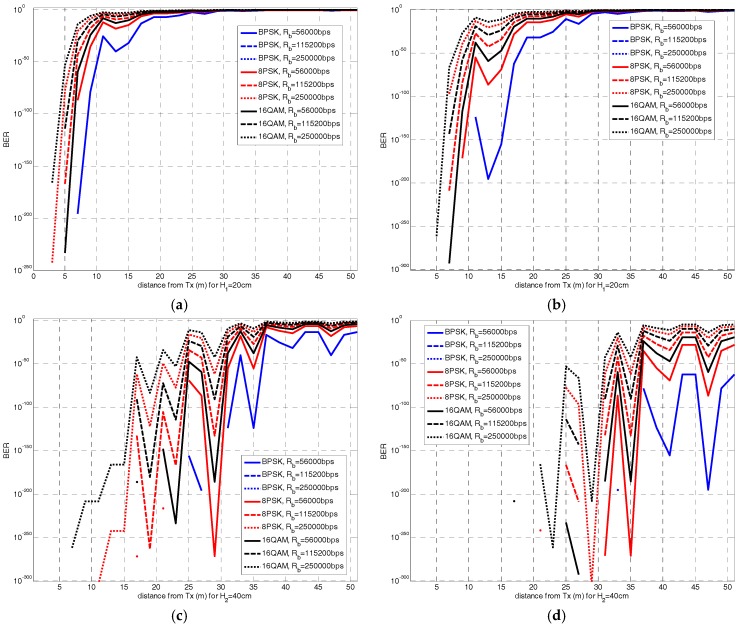
BER for (**a**) *H*_1_ and *B* = 100 MHz; (**b**) *H*_1_ and *B* = 100 MHz; (**c**) *H*_2_ and *B* = 20 MHz; (**d**) *H*_2_ and *B* = 20 MHz.

Figure 6a–d shows the calculation of BER for the two antenna heights *H*_1_ and *H*_2_ and a combination of the parameters that define BER: three values of data rate $R_{b}$ (56 Kbps, 115.2 Kbps and 250 Kbps), two values of power noise $N_{0}$ for bandwidths of *B* = 100 MHz (full 2.4 GHz band) and *B* = 20 MHz (channel bandwidth for the 2.4 GHz band), and number of symbols *M* (BPSK, 8PSK and 16QAM).

A high variability is observed between the different cases, with higher values of BER achieved for more complex modulation schemes. Additionally, it is noticed the difference between the considered data rates, leading to a lower BER for the lowest data rate values. Finally, as *N*_0_ is higher (larger bandwidth *B*), the BER values are lower.

It is also observable that the cases for *H*_1_ show a BER value under 10^−6^ if the distance from transmitter stays under 20–25 m. An 802.11 certified device must carry out a receiver sensitivity threshold of −83 dBm and BER of 10^−4^. For the antenna height *H*_2_ this inflection point shows up at a further distance, over 35 m.

Then, we can state that the near-ground scenario is more restrictive than an OLoS link, and would require the use of more robust modulations or error correction codes to equal the performance of LoS based networks. These results become helpful for optimization of the design and deployment of a near-ground WSN depending on the involved parameters: modulation, data rate and the level of *N*_0_.

## 4. Discussion

Near-ground models proposed in the literature are scarce given that most actual radio channels are designed to be far above the ground [24,26]. It has been known that lowering the antennas height would significantly decrease the signal strength [17,25], therefore reducing the system range. This effect was described in [17] proposing a two-slope log-distance model for the path loss occurring in a WSN at 868 MHz in an open area.

In Table 3 some models and experimental results reported in literature are briefly described, showing their scope and validity. The main conclusion to infer is that near-ground models require experimental measurements in order to validate the main propagation mechanisms occurring. Simulation-based solutions present limited scalability and remains restricted to specific environments and certain requirements [17,24,25,26,27,28,29,30,31,32].

**Figure 7:**
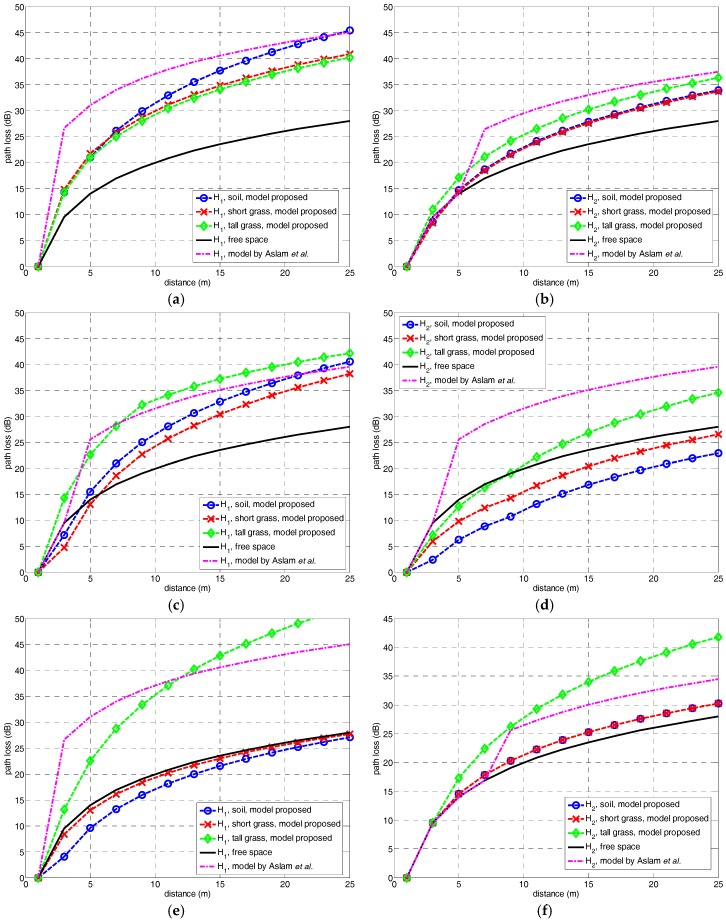
Comparison of path loss models, at (**a**) $F_{1}$, $H_{1}$; (**b**) $F_{1}$, $H_{2}$; (**c**) $F_{2}$, $H_{1}$; (**d**) $F_{2},\;H_{2}$; (**e**) $F_{3},\;H_{1}$; and (**f**) $F_{3}$, $H_{2}$.

In the plots of Figure 4a, a basic comparison was performed between the measurement results, the proposed model and the free-space attenuation. Among the mentioned existing models, the one given in [23] was chosen as the closer approach to perform a comparison. Figure 7 shows a comparison between the proposed model, free-space and the model in [23]. This approach determines the path loss $P_{L}$ as a two-slope log-normal trend dependent of the link range, according to the expressions in (14) and (15):

(14) $$\begin{array}{cc} P_{L}\left(d\right)=L_{fs} & ,\;d<\;d_{b} \end{array}$$

(15) $$\begin{array}{cc} P_{L}\left(d\right)=L_{fs}+L_{NG} & ,\;d\geq \;d_{b} \end{array}$$

with $L_{fs}$ the free space path loss, $L_{NG}$ is the near-ground path loss in dB and $d_{b}$ is the minimum distance for the existence of near-ground path loss.

The near-ground path loss $L_{NG}$ is obtained as indicated in (16), also in dB:

(16) $$\begin{array}{c} L_{NG}=-20\cdot log_{10}\left(\frac{5\sqrt{h_{t}h_{r}}}{3\sqrt{d\lambda }}+\frac{35h_{t}h_{r}}{6d\lambda }\right) \end{array}$$

where respectively $h_{t}$ and $h_{r}$ are the heights of the transmitter and the receiver and *λ* is the wavelength [16].

The minimum distance for the existence of near-ground path loss is defined by (17):

(17) $$\begin{array}{c} d_{b}=\frac{h_{t}h_{r}}{0.09\lambda } \end{array}$$

This expression is derived from assuming that the FFZ is obstructed in a 30% which represents half of the requirement for the existence of diffraction loss; in other words, the clearance *h* meets the expression in (18):

(18) $$\begin{array}{cc} h\leq \frac{1}{2}\cdot 0.6\cdot r_{1}=0.3\cdot \sqrt{d\lambda }\to & \begin{array}{cc} d\geq \frac{h}{0.09\lambda }\to & d_{b}=\frac{h}{0.09\lambda } \end{array} \end{array}$$

For a more general case in which the transmit and receive antennas have different heights, h is replaced by $h_{t}h_{r}$.

Generally speaking, from the comparison in Figure 6 an over-estimation of the path loss maybe concluded, except for the tall grass case. An explanation may be found in the low effective antenna height of this type of ground. The model proposed in this paper seems to be more realistic for scenarios.

Other recent models can be found, and even that not comparable to our case, the analysis used may result in interest for future on-going research: study over irregular terrain in 200–600 MHz [31], forest at 2.4 GHz [32], snow covered forest at 2.45 GHz [33], a ray-tracing tool for simulation [34] or a UWB near-ground wireless network [35].

## 5. Conclusions

As any radio channel, both narrowband and wideband characterization are required to determine the propagation properties of a near-ground scenario. From the wideband characterization the channel parameters obtained are power delay and angular profiles, RMS delay and angular spread, coherence bandwidth, and coherence distance. The path loss can be derived of both narrowband channel characterization. The narrowband model becomes a critical tool for the design of the wireless network in terms of system range and quality of service.

In this paper, a narrowband radio channel model is proposed for a scenario with the radio link operating under near-ground conditions and using directional antennas, applied to a smart agriculture use case in bands planned for 5G-IoT. A comparison of the path loss results for different combinations of antenna heights, frequency bands and ground coverings demonstrated the influence of those elements:

1. The difference between the free-space and measured path loss decreases as the transmitting frequency increases due to a larger obstruction of the FFZ. Because of this, the 868 MHz seems not to be an optimal solution just for minimizing path losses.
2. Lowering the height of the antennas produces a larger signal attenuation also explained by a larger obstruction of the FFZ.
3. The height of the ground covering (grass leaf length, p.e.) reduces the effective antenna height so increasing the obstruction of the FFZ.

The path loss was determined to be dependent of the radio link range according to a three-slope log-distance model. The characteristics of the near-ground radio channel may be mainly explained in terms of Fresnel zones obstruction, and the proposed model reflects this fact. Actually, the main contribution of the approach here described is to enlighten that the distance between the transmitter and the receiver may be split into three zones limited by two critical points and the break or cross-over point.

The QoS analysis performed for this case, derived from RSSI experimental values along a distance of 51 m, leads to state that the close ground scenario is more restrictive than an OLoS link, and it may require the use of more robust digital modulation schemes or error correction codes to equal the performance of LoS based networks.

A near-ground setup can show up in a WSN even when the network configuration was not originally planned as such, p.e. in case of grass or crops growth occurs. Then, the network should be provided of any type of intelligence to prevent, correct or control such situation, so that a more proper modulation can be chosen, or data routing can be adapted to avoid those paths. An option would be to include software agents [36] dedicated to the mission of monitoring the radio channel obstruction.

Even near-ground networks are increasingly used; many studies proposed in the literature lack generality and flexibility to be tested in a different scenario. The study and characterization of these systems admits a deep and extensive study to respond to the plausible influence of variables, such as the scenario, frequency bands, antenna radiation pattern and polarization, terrain roughness and profile, ground composition and dielectric properties or the fixed antenna height [15,16,17,18,19,20,21,22].

Also missing is the application of techniques for increasing the capacity of the near-ground casuistry, such as diversity or MIMO. Only one work was found related to the analysis of MIMO to improve the effect of the diffraction topic [16]. Among other likely research lines, the design of ad-hoc antennas constitutes a goal for these systems, with a radiation pattern directive and with an elevation that minimizes the FFZ obstruction.

The result of achieving a more accurate characterization, and standardization, of the near-ground systems would increase its use remarkably, broadening the variety of use cases, mainly in the short-term future of 5G-IoT.

## Figures and Tables

**Table 1:** Obstruction of the First Fresnel Zone (FFZ) in percentage w.r.t antenna height.

	$F_{1}\;=\;868\;\mathrm{MHz}$	$F_{1}\;=\;2.4\;\mathrm{GHz}$	$F_{1}\;=\;5.8\;\mathrm{GHz}$
$r_{1}=\sqrt{d\lambda }$ (m)	2.94	1.77	1.14
% $H_{1}/r_{1}$	93.2	88.7	82.4
% $H_{2}/r_{2}$	86.4	77.4	64.8

**Table 2:** Attenuation factors for the path loss model proposed in (5)–(7).

		$F_{1}=868\;\mathrm{MHZ}$			$F_{2}=2.4\;\mathrm{GHZ}$			$F_{3}=5.8\;\mathrm{GHZ}$		
		$n_{1}$	$n_{2}$	$n_{3}$	$n_{1}$	$n_{2}$	$n_{3}$	**#1**	**#2**	**#3**
**SOIL**	$H_{1}$	3	3.5	3.5	1.5	3.75	3.5	0.85	2.6	2.6
	$H_{2}$	1.8	2.75	2.75	0.5	1.75	2.75	2	2.25	2.6
**SHORT** **GRASS**	$H_{1}$	3.1	2.75	3.5	1.0	3.75	3.5	1.75	2.1	2.6
	$H_{2}$	1.75	2.75	2.75	1.25	1.75	2.75	2	2.25	2.6
**TALL** **GRASS**	$H_{1}$	3	2.75	2.75	3	3.75	2.25	2.75	4.25	2.6
	$H_{2}$	2.3	2.75	2.75	1.5	2.5	3.5	2	3.5	2.6

**Table 3:** Description of models and experimental results reported for near-ground radio channels.

Reference	Description
[16]	Theoretical model and ray-tracing simulation for a near-ground channel at mmw band. Analysis of diffuse scattering in MIMO applied to near-ground channel.
[25]	Results of RSSI measurements with distance for a WSN in indoors, outdoors, different blocking conditions and antenna elevations. Only flat floors. Unknown frequency band used. *Not a model*.
[17]	Two-slope log-distance model for the path loss for a WSN at 868 MHz in an open area.
[24,25,26,27,28,29,30,31,32]	Software simulation of limited scalability only valid for certain environments.
[18]	Analysis of impact of foliage on near-ground radiowave propagation for battlefield sensor networks operating at 300 MHz and 1900 MHz. *Not a model*.
[28]	Measurement results for ground-based UHF band communicators in urban terrain for both line-of-sight (LOS) and non-line-of-sight (NLOS) links. *Not a model*.
[23,29]	Numerical solver-based simulation for near-ground long range propagation; computational complexity limits a large number of nodes in the simulated network. *Not a model*.
[30]	Simple mathematical path loss model; overlooks the significant impact of terrain roughness and electrical properties on the channel transfer characteristics.
[23]	Theoretical two-slope path model based on the condition of 50% of obstruction for the first Fresnel zone.
